# Efficacy of Povidone Iodine Against Microbial Biofilms in Breast Implants With Different Textures: Results From an *in vitro* Study

**DOI:** 10.3389/fmicb.2022.868347

**Published:** 2022-03-29

**Authors:** Borja Fernández-Ibarburu, Marta Díaz-Navarro, Gorka Ibarra, Andrés Rivera, Rama Hafian, Ãlvaro Irigoyen, Raquel Carrillo, Rosa Pérez-Cano, Patricia Muñoz, Ángela García-Ruano, José M. Lasso, María Guembe

**Affiliations:** ^1^Department of Plastic Surgery, Hospital General Universitario Gregorio Marañón, Madrid, Spain; ^2^Instituto de Investigación Sanitaria Gregorio Marañón, Madrid, Spain; ^3^Department of Clinical Microbiology and Infectious Diseases, Hospital General Universitario Gregorio Marañón, Madrid, Spain; ^4^CIBER Enfermedades Respiratorias-CIBERES (CB06/06/0058), Madrid, Spain; ^5^Medicine Department, School of Medicine, Universidad Complutense de Madrid, Madrid, Spain

**Keywords:** biofilm, breast implant, *in vitro* model, irrigating solutions, pocket irrigation, povidone iodine

## Abstract

**Background:**

In the practice of breast augmentation and reconstruction, implant irrigation with various solutions has been widely used to prevent infection and capsular contracture, but to date, there is no consensus on the optimal protocol to use. Recently, application of povidone iodine (PI) for 30 min has shown *in vitro* to be the most effective irrigating formula in reducing contamination in smooth breast implants. However, as 30 min is not feasible intraoperatively, it is necessary to determine whether shorter times could be equally effective as well as to test it in both smooth and textured implants.

**Methods:**

We tested the efficacy of 10% PI at 1′, 3′, and 5′ against biofilms of 8 strains (2 ATCC and 6 clinical) of *Staphylococcus spp*. on silicone disks obtained from Mentor^®^ and Polytech^®^ implants of different textures. We analyzed the percentage reduction of cfu counts, cell viability and bacterial density between treatment (PI) and control (sterile saline, SS) groups for each time of application. We consider clinical significance when > 25% reduction was observed in cell viability or bacterial density.

**Results:**

All textured implants treated with PI at any of the 3 exposure times reduced 100% bacterial load by culture. However, none of the implants reached enough clinical significance in percentage reduction of living cells. Regarding bacterial density, only 25–50 μm Polytxt^®^ Polytech^®^ implants showed significant reduction at the three PI exposure times.

**Conclusion:**

PI is able to inhibit bacterial growth applied on the surface of breast implants regardless of the exposure time. However, no significant reduction on living cells or bacterial density was observed. This lack of correlation may be caused by differences in texture that directly affect PI absorption.

## Introduction

In the field of Plastic Surgery, the use of breast implants (BI) is a fundamental pillar, both in reconstructive and aesthetic surgery. According to the International Society of Aesthetic Plastic Surgery (ISAPS), every year nearly two million women undergo breast augmentation with implants throughout the world; in Spain the number reaches 50,000 women operated on annually, compared to over 400,000 women a year in the United States (ISAPS, 2019).

In breast surgery with implants, infection of the surgical site as a consequence of bacterial colonization of the BI is one of the most relevant complications. While in cosmetic breast augmentation its incidence is around 0.1–1.5%, it rises to 5.8–28% in breast reconstruction, depending on the study (ISAPS, 2019). Consequences of these infections are a delay in starting adjuvant chemotherapy or radiotherapy treatment and additional surgeries with a significant increase in the risk of reconstructive failure and/or implant loss (Ranganathan et al., 2018; Ngaage et al., 2020).

In the context of BI colonization by pathogenic microorganisms, the presence of bacterial biofilm has been correlated with the development of capsular contracture, which is the most frequent complication in breast implant surgery (Drinane et al., 2017). Such bacterial contamination of the implant shell has also been proposed as one of the factors involved in the pathogenesis of breast implant-associated anaplastic large cell lymphoma (BI-ALCL) (Jones et al., 2018).

In relation to the type of breast implants (shell architecture), multiple studies have shown that textured BI have higher rates of bacterial growth *in vitro*, compared to those with a lower degree of surface texturing (Jones et al., 2018; James et al., 2019). However, no consistent association has been demonstrated between smooth or textured implants and capsular contracture, but has so with the severity (Poeppl et al., 2007). In general, textured implants are preferable because they have a lower rate of pocket over-dissection, rotation and displacement, and they adhere more to the surrounding tissues. Smooth implants have greater long-term complications such as seroma and capsular contracture.

As for BI colonizing microorganisms, the most frequently isolated in implant culture is Staphylococci (*S. epidermidis* and *S. aureus*) and anaerobes, which are associated with treatment-resistant infection and implant loss (Del Pozo et al., 2009; Deva et al., 2013; Rieger et al., 2013; del Pozo and Auba, 2015; Ngaage et al., 2020).

Several therapeutic measures have been proposed to reduce the bacterial load of implants during surgery, such as washing the breast pocket with antiseptic solutions, being the most common a triple antibiotic dilution (Cefazolin 1 g, Bacitracin 50,000 U, Gentamicin 80 mg) followed by povidone iodine (PI) (Chopra et al., 2017; Drinane et al., 2017; Banerjee and Featherstone, 2019; Carvajal et al., 2019). Another alternative is to irrigate or immerse the implant before its placement; but to date there is no agreement on the optimal product to use (Ta et al., 2008; Dang et al., 2020). Despite this lack of consensus, a recent study by Ngaage et al. (2020) has shown that PI is the most effective irrigating formula in reducing contamination of smooth breast implants by methicillin-resistant *S. aureus* and *S. epidermidis*, shown in a 30-min *in vitro* test. However, in surgery, it is not feasible to wait 30 min for the solution to act, as the implants cannot be submerged during surgery for that time. Despite usually implants are pre-selected, the final choice is made at the end of the surgery, when the dissection of the prosthetic pocket is completed; so waiting an additional 30 min would mean such a prolongation of the surgery time, leading to increased costs and morbidity.

So it is necessary to determine whether shorter times could be equally effective, as it has been demonstrated in other fields (Ta et al., 2008).

In this regard, given that in Spain the majority of implants used are textured, we determined using an *in vitro* model of the efficacy of 10% PI applied for 3 different time lengths, against bacterial biofilm formation of *S. aureus* and *S. epidermidis* strains on breast implants with different degrees of texture.

## Materials and Methods

### Setting

The study was performed in the Microbiology Laboratory of Hospital General Universitario Gregorio Maranon, a tertiary institution in Madrid, Spain.

### Laboratory Procedure

We performed an *in vitro* model with silicone implant disks based on a two-phase model with a pre-treatment step (disinfection) with either PI or sterile saline (SS, for positive controls), followed by a contamination step with bacterial suspensions to assess the impact on colony forming unit (cfu) counts, cell viability rate, and bacterial density.

#### Strains

We selected 4 strains of coagulase-negative Staphylococci and 2 strains of *S. aureus* isolated from breast implants samples which previously demonstrated to be high biofilm producers (by crystal violet assay), archived in the Microbiology Service corresponding to the MICRO.HGUGM-2016-027 project. We also included 2 ATCC strains: *S. epidermidis* ATCC 35884 and methicillin-susceptible *S. aureus* ATCC 29213.

**FIGURE 1:**
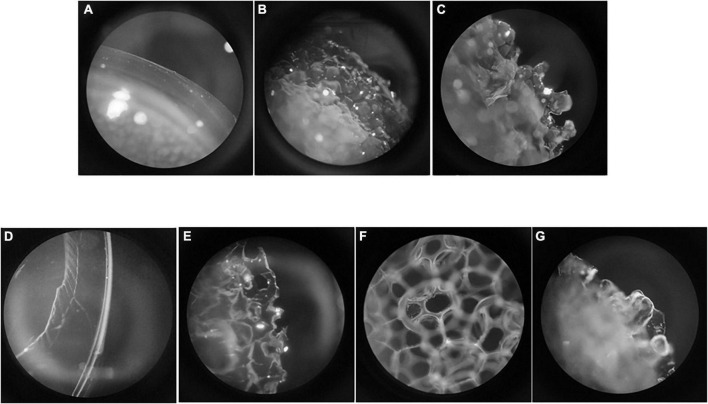
Surface of each implant. **(A)** Mentor® smooth (10 μm); **(B)** Mentor® Siltex™ (28 μm); **(C)** Mentor® CPG™ (36 μm); **(D)** Polytech® Polysmooth™ (< 10 μm); **(E)** Polytech® MESMO® (25 μm); **(F)** Polytech® Polytxt® (25–50 μm); **(G)** Polytech® macrotextured (50–100 μm).

The following implants of different textures from two suppliers were used (Figure 1):

Mentor® (Santa Barbara, CA): 10 μm (smooth), 28 μm (Siltex™, microtextured), 36 μm (CPG™, microtextured).

Polytech® (Dieburg, Germany): < 10 μm (Polysmooth™), 25 μm (MESMO^®^, microtextured), 25–50 μm (Polytxt^®^, microtextured), 50–100 μm (macrotextured).

From each implant, 6 mm diameter disks were prepared using a punch. The disks were sterilized by ethanol immersion followed by autoclaving (121°C, 15 min) before use.

#### Methodology

**FIGURE 2:**
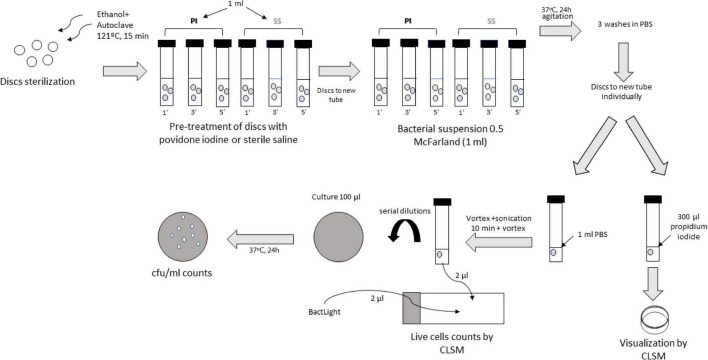
Laboratory procedure.

The two-phase model was based on a pre-treatment step (disinfection) followed by a contamination step, which was carried out by immersing disks in glass tubes at a ratio of 1 disk/300 μl of medium. Sonication was carried out in 1 ml of phosphate buffered saline (PBS) (Figure 2).

Experiments were all carried out in triplicate.

1. Disinfection step: Immersion of the disks (3 by 3) in glass tubes containing 900 μl of a 10% PI solution for either 1, 3, or 5 min. In parallel, the same procedure was carried out with 0.9% SS as a positive control. The negative control was treated with PI for 1 min.
2. Contamination step: After adsorption step, wet disks were immediately placed in new glass tubes containing 900 μl of bacterial suspensions (0.5 McFarland = 2.5 × 10^8^ cfu/ml) of each strain in PBS. The negative control was inoculated with only PBS. Tubes were incubated at 37°C and agitated for 24 h. After this period, the disks were washed 3 times with PBS to remove unattached bacteria. The model was performed in order to mimic a clinical scenario, so contamination step was performed by transferring discs “treated” with PI or saline to the bacterial solution, considering that PI has act during disinfection step at the different exposure times, so no PI is longer needed.
3. Sessile cell recovery: After washing the disks, they were individually transferred to new glass tubes containing 1 ml PBS, sonicated for 10 min at 40 kHz to detach the biofilm. The sonicated bacterial suspension was then vortexed for culture and confocal laser scanning microscopy (CLSM).
4. Culture of sonicate: After vortexing the sonicated bacterial suspension, one part was serially diluted and 100 μl of dilutions were cultured on blood agar plates. Plates were incubated at 37°C for 24 h and cfu/plate counts were performed.

For bacterial density analysis, experiments were performed in the same way, but using one disk per strain and treatment (instead of triplicates). After the contamination step, disks were directly transferred individually to a new glass tube containing 300 μl of propidium iodide for subsequent visualization of the biofilm thickness/depth by CLSM exploring 3 fields in each disk.

#### Variables

1. cfu count: Based on cfu/plate counts, cfu/ml were calculated and expressed on a logarithmic scale.
2. Cell viability rate: 2 μl of the sonicated bacterial suspension was stained on slides with 0.2 μl BacLight® (composed of Syto 9, which stains living and dead cells in green, and propidium iodide, which only stains dead cells in red) for subsequent visualization by CLSM. Three fields were explored and the rate of percentage of living cells was calculated as follows: living cells/ (living + dead cells) × 100.
3. Bacterial density: Three fields per sample were obtained by CLSM and bacterial density was calculated as no. bacteria/μm^2^.

First, the disks were washed 3 times with PBS to eliminate planktonic cells, and after stained for 10 min with propidium iodide using 0.5% Triton-X 100 and 4% formaldehyde to obtain an image of 24 h biofilm growth accumulation on surfaces. Propidium iodide-stained cells were examined using a Leica TCS SPE confocal fluorescence microscope (Leica Geosystems AG, Heerbrugg, Switzerland). The biofilm depth was measured at 4 μm intervals from the bottom of the biofilm along 80 μm with a 10x objective. Finally, images were processed using FIJI (Image J) software (National Institute of Health, Bethesda, MD). Data of bacterial density was estimated at the stacks maximum z-projections.

#### Data Analysis

From each and every experiment the median (IQR) of log cfu/ml, percentage of living cells, and bacterial density was calculated.

To characterize the efficacy of the three PI exposure times relative to the positive control (sterile saline), mean % reduction in log cfu/ml, % reduction in living cells, and % reduction in bacteria/μm^2^ of biofilm was calculated. This was also calculated according to the implant brand, texture, and microorganism.

#### Statistical Analysis

Quantitative variables are expressed as the median and interquartile range (IQR). We used parametric methods (*t* or ANOVA) or non-parametric methods (median test). Linear or logistic regression models were fitted in cases of asymmetry.

Statistical significance was set at *p* < 0.05 for all the tests. The statistical analysis was performed using IBM SPSS Statistics for Windows, Version 21.0 (IBM Corp, Armonk, NY) and Software GraphPad Prism 7.0 (San Diego, CA).

We also defined clinical significance when mean percentage reduction cfu counts, living cells and bacterial density reached at least 25% for PI treatment in comparison to SS.

### Availability of Data and Material

Datasets will be kept by the Microbiology and Infectious Diseases Service and the data collection will be registered in the repository of the Instituto de Salud Carlos III (ISCIII) under number C.0001228.

## Results

### Mentor® Implants

**FIGURE 3:**
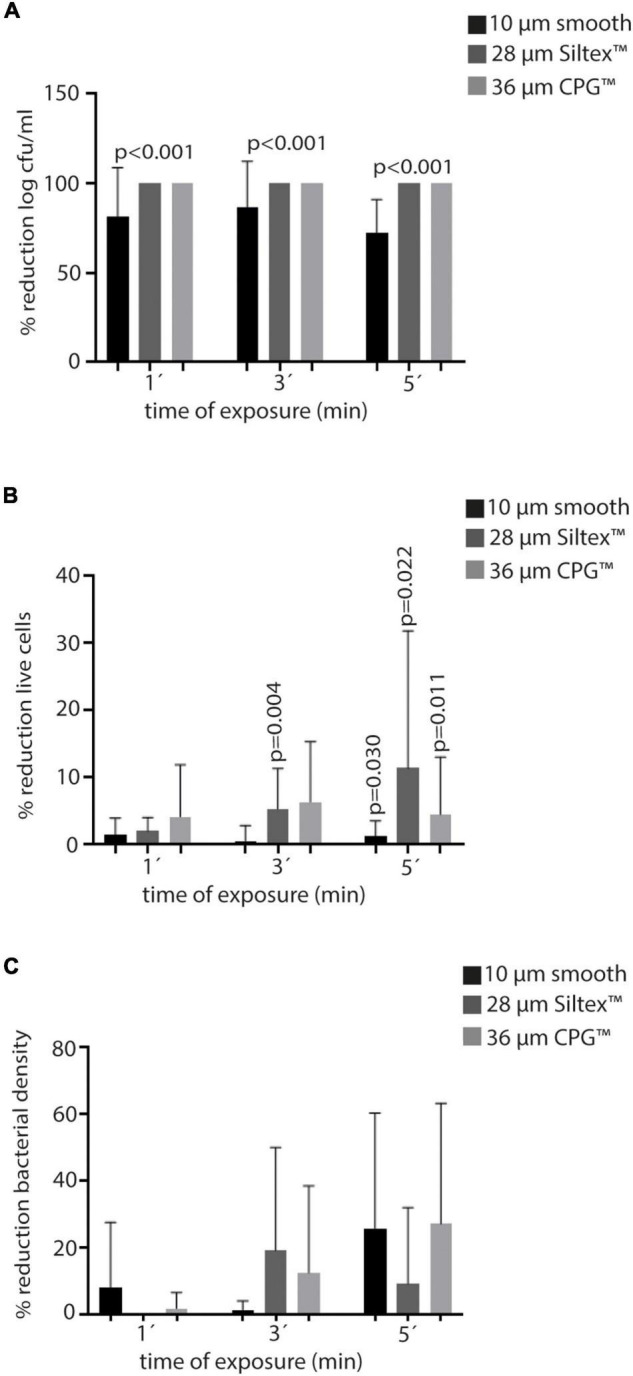
Efficacy of PI in Mentor® at the three times of exposure (1′, 3′, and 5′) in terms of mean percentage reduction of the variables of all Staphylococci. **(A)** Log cfu/ml. **(B)** Live cells. **(C)** Bacterial density. PI, povidone iodine; cfu, colony forming units.

Overall, we found clinically and statistically significant differences between PI and SS at each time of exposure in all microorganisms tested with every type of implant. We did not obtain any cfu cultures from the disks sonicated at PI1′, PI3′, and PI5′ except for the 10 μm—smooth texture, where the median (IQR) log cfu/ml for PI1′, PI3′, and PI5′ was, respectively: 0.00 (0.00–1.35), 0.00 (0.00–0.33), and 1.00 (0.00–1.50) (Table 1, Figures 3A–C, and Supplementary Figures 8–10).

**TABLE 1:** Results for povidone iodine at three times of exposure in Mentor® implants.

Prosthesis	Treatment	Median (IQR) log ufc/ml	*p* *	Median (IQR) % live cells	*p* *	Median (IQR) % bacterial density	*p* *
**10 μm smooth**	SS 1′	3.33 (2.89–4.42)	**<0.001**	100.00 (100.00–100.00)	0.531	0.04 (0.01–0.07)	0.010
	PI 1′	0.00 (0.00–1.35)		100.00 (100.00–100.00)		0.09 (0.06–0.12)	
	SS 3′	3.65 (3.06–4.09)	**<0.001**	100.00 (100.00–100.00)	0.272	0.05 (0.01–0.07)	0.198
	PI 3′	0.00 (0.00–0.33)		100.00 (96.40–100.00)		0.07 (0.02–0.11)	
	SS 5′	3.10 (2.98–4.63)	**<0.001**	100.00 (100.00–100.00)	0.052	0.07 (0.05–0.08)	0.775
	PI 5′	1.00 (0.00–1.50)		100.00 (94.78–100.00)		0.07 (0.03–0.12)	
**28 μm Siltex™**	SS 1′	4.77 (4.22–5.35)	**<0.001**	100.00 (99.40–100.00)	0.097	0.12 (0.06–0.21)	0.004
	PI 1′	0.00 (0.00–0.00)		100.00 (94.50–100.00)		0.25 (0.18–0.33)	
	SS 3′	4.88 (4.54–5.21)	**<0.001**	100.00 (95.30–100.00)	**0.004**	0.18 (0.06–0.51)	0.392
	PI 3′	0.00 (0.00–0.00)		95.90 (88.35–98.00)		0.31 (0.14–0.50)	
	SS 5′	4.85 (4.17–4.99)	**<0.001**	100.00 (94.10–100.00)	0.346	0.10 (0.09–0.12)	0.153
	PI 5′	0.00 (0.00–0.0)		99.00 (78.38–100.00)		0.17 (0.06–0.21)	
**36 μm CPG™**	SS 1′	3.98 (3.10–4.75)	**<0.001**	100.00 (97.68–100.00)	**0.030**	0.06 (0.03–0.16)	0.001
	PI 1′	0.00 (0.00–0.00)		96.45 (90.65–100.00)		0.19 (0.14–0.23)	
	SS 3′	4.02 (3.29–4.75)	**<0.001**	100.00 (98.48–100.00)	**0.022**	0.09 (0.06–0.18)	0.253
	PI 3′	0.00 (0.00–0.00)		95.95 (86.78–100.00)		0.14 (0.08–0.18)	
	SS 5′	3.88 (3.00–4.89)	**<0.001**	100.00 (99.50–100.00)	**0.011**	0.15 (0.11–0.15)	0.775
	PI 5′	0.00 (0.00–0.00)		97.00 (93.20–100.00)		0.14 (0.07–0.17)	

*SS, sterile saline; PI, povidone iodine; IQR, interquartile range; cfu, colony forming units; bacterial density, bacteria/μm^2^.*

*Values in bold represent statistical significance in favor of PI.

Regarding cell viability, expressed in percentage of living cells recovered from the disk sonication, none of the treatments with PI showed more than 15% mean reduction compared to SS. However, in some circumstances, statistical significance was achieved when median values were compared (Table 1, Figures 3A–C, and Supplementary Figures 8–10).

Median bacterial density was higher in PI treatments than in SS at every time of exposure in all Mentor® implants. Only 36 μm CPG™ implants showed a significant mean percentage reduction of bacterial density at PI 5′ (31.6%) (Figure 3C), and 10 μm—smooth implants treated with PI5′ showed a lower median bacterial density (*S. epidermidis*), compared with those treated only with SS (*p* = 0.035) (Supplementary Figures 8–10).

#### Comparison

Data of the comparison between each type of Mentor® implants is detailed in Table 2. 28 μm Siltex™ and 36 μm CPG™ implants showed a higher reduction of cfu count compared to 10 μm—smooth implants after PI treatment.

**TABLE 2:** Comparative results for povidone iodine at three times of exposure according to Mentor® implants.

	Treatment	Median (IQR) log ufc/ml	*p* *	Median (IQR) % live cells	*p* *	Median (IQR) % bacterial density	*p* *
**28 μm Siltex™** vs. **36 μm CPG™**	**PI 1′ 28 μm Siltex™**	0.00 (0.00–0.00)	1	100.00 (94.50–100.00)	0.262	0.25 (0.18–0.33)	**0.087**
	**PI 1′ 36 μm CPG™**	0.00 (0.00–0.00)		96.45 (90.65–100.00)		0.19 (0.14–0.23)	
	**PI 3′ 28 μm Siltex™**	0.00 (0.00–0.00)	1	95.90 (88.35–98.00)	0.596	0.31 (0.14–0.50)	**0.001**
	**PI 3′ 36 μm CPG™**	0.00 (0.00–0.00)		95.95 (86.78–100.00)		0.14 (0.0–0.18)	
	**PI 5′ 28 μm Siltex™**	0.00 (0.00–0.00)	1	99.00 (78.38–100.00)	0.934	0.17 (0.06–0.21)	0.253
	**PI 5′ 36 μm CPG™**	0.00 (0.00–0.00)		97.00 (93.20–100.00)		0.14 (0.07–0.17)	
**28 μm Siltex™** vs. **10 μm smooth**	**PI 1′ 28 μm Siltex™**	0.00 (0.00–0.00)	**0.004**	100.00 (94.50–100.00)	**0.041**	0.25 (0.18–0.33)	**<0.001**
	**PI 1′ 10 μm smooth**	0.00 (0.00–1.35)		100.00 (100.00–100.00)		0.09 (0.06–0.12)	
	**PI 3′ 28 μm Siltex™**	0.00 (0.00–0.00)	**0.037**	95.90 (88.35–98.00)	**0.002**	0.30 (0.14–0.50)	**<0.001**
	**PI 3′ 10 μm smooth**	0.00 (0.00–0.33)		100.00 (96.40–100.00)		0.07 (0.02–0.11)	
	**PI 5′ 28 μm Siltex™**	0.00 (0.00–0.00)	**<0.001**	99.00 (78.38–100.00)	0.120	0.17 (0.06–0.21)	**0.032**
	**PI 5′ 10 μm smooth**	1.00 (0.00–1.50)		100.00 (94.78–100.00)		0.07 (0.03–0.12)	
**10 μm smooth** vs. **36 μm CPG™**	**PI 1′ 10 μm smooth**	0.00 (0.00–1.35)	**0.004**	100.00 (100.00–100.00)	**0.004**	0.09 (0.06–0.12)	**0.001**
	**PI 1′ 36 μm CPG™**	0.00 (0.00–0.00)		96.45 (90.65–100.00)		0.19 (0.14–0.23)	
	**PI 3′ 10 μm smooth**	0.00 (0.00–0.33)	**0.037**	100.00 (96.40–100.00)	**0.036**	0.07 (0.02–0.11)	**0.002**
	**PI 3′ 36 μm CPG™**	0.00 (0.00–0.00)		95.95 (86.78–100.00)		0.14 (0.08–0.18)	
	**PI 5′ 10 μm smooth**	1.00 (0.00–1.50)	**<0.001**	100.00 (94.78–100.00)	0.072	0.07 (0.03–0.12)	**0.087**
	**PI 5′ 36 μm CPG™**	0.00 (0.00–0.00)		97.00 (93.20–100.00)		0.14 (0.07–0.17)	

*PI, povidone iodine; IQR, interquartile range; cfu, colony forming units; bacterial density, bacteria/μm^2^.*

*Values in bold represent statistical significance.

Reduction of median (IQR) of living cells was imperceptible between the three types, and statistical significance was found in PI1′ and PI3′ between 28 μm Siltex™ vs. 10 μm—smooth implants.

Regarding bacterial density, 10 μm—smooth implants showed a better reduction than 28 μm Siltex™ and 36 μm CPG™ (Supplementary Figures 1–3).

**FIGURE 4:**
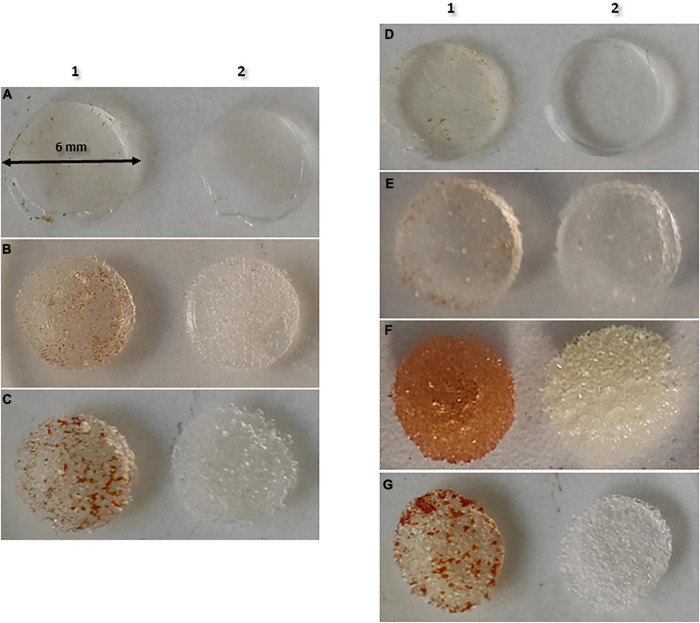
Absorption of PI in each type of implant. **(A)** Mentor® smooth (10 μm); **(B)** Mentor® Siltex™ (28 μm); **(C)** Mentor® CPG™ (36 μm); **(D)** Polytech® Polysmooth™ (<10 μm); **(E)** Polytech® MESMO® (25 μm); **(F)** Polytech® Polytxt® (25–50 μm); **(G)** Polytech® macrotextured (50–100 μm). **1**, povidone iodine; **2**, saline.

As showed in Figures 4A–C, the degree of PI absorption was different depending on the type of Mentor® implant, being the 36 μm CPG™ implant the one with the highest degree of absorption.

### Polytech® Implants

**FIGURE 5:**
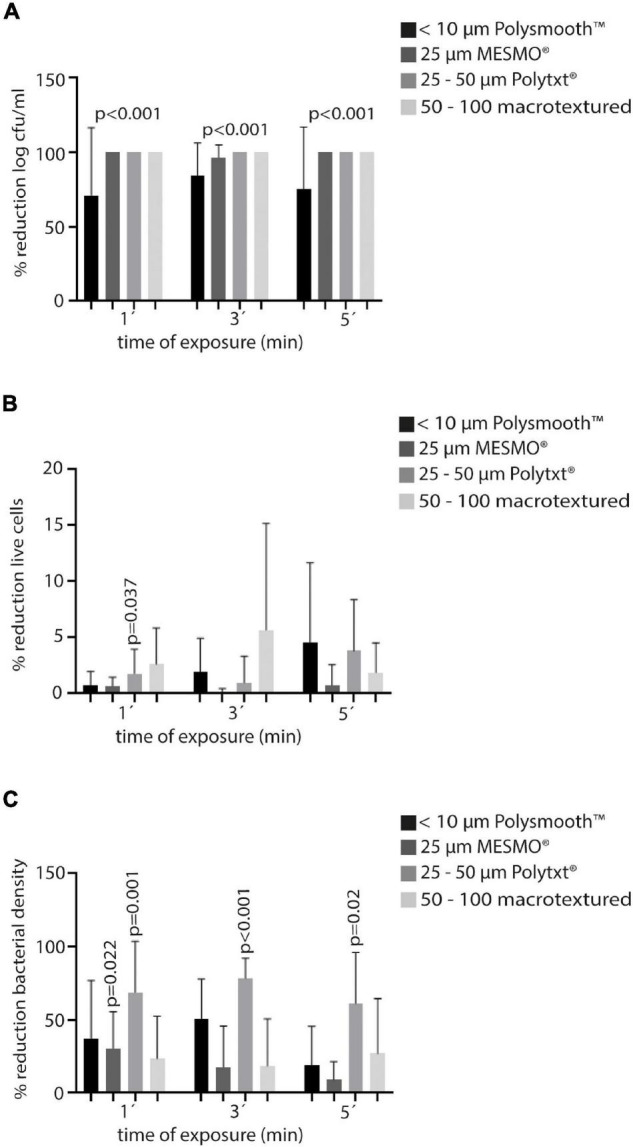
Efficacy of PI in Polytech® at the three times of exposure (1′, 3′, and′) in terms of mean percentage reduction of the variables of all Staphylococci. **(A)** Log cfu/ml. **(B)** Live cells. **(C)** Bacterial density. PI, povidone iodine; cfu, colony forming units.

As what was shown with Mentor® implants, statistical and clinical significance was observed between median cfu counts recovered in all Polytech® implants treated with PI compared to control, at every time of exposure. No bacteria were recovered in sonicated disks of 25 μm MESMO®, 25–50 μm Polytxt® or 50–100 μm macrotextured implants treated with PI (mean % reduction rates for PI1′, PI3′, and PI5′ was 100% each). However, the same results were not observed in < 10 μm Polysmooth™ implants, where the mean % reduction rates for PI1′, PI3′, and PI5′ was, respectively: 65.3, 82.9, and 51.9% (Table 3, Figures 5A–C, and Supplementary Figures 11–13).

**TABLE 3:** Results for povidone iodine at three times of exposure in Polytech® implants.

Prosthesis	Treatment	Median (IQR) log ufc/ml	*p* *	Median (IQR) % live cells	*p* *	Median (IQR) % bacterial density	*p* *
**<10 μm Polysmooth™**	SS 1′	2.67 (2.33–3.16)	**<0.001**	100.00 (100.00–100.00)	0.599	0.20 (0.12–0.35)	0.153
	PI 1′	0.00 (0.00–2.23)		100.00 (100.00–100.00)		0.12 (0.07–0.29)	
	SS 3′	2.78 (2.53–3.15)	**<0.001**	100.00 (100.00–100.00)	0.752	0.22 (0.08–0.39)	0.392
	PI 3′	0.00 (0.00–1.72)		100.00 (100.00–100.00)		0.12 (0.04–0.20)	
	SS 5′	2.82 (1.94–3.13)	**<0.001**	100.00 (98.73–100.00)	0.270	0.18 (0.11–0.20)	0.392
	PI 5′	0.00 (0.00–1.51)		100.00 (89.90–100.00)		0.19 (0.12–0.30)	
**25 μm MESMO®**	SS 1′	3.70 (3.61–3.96)	**<0.001**	100.00 (100.00–100.00)	0.339	0.17 (0.14–0.19)	**0.022**
	PI 1′	0.00 (0.00–0.00)		100.00 (100.00–100.00)		0.11 (0.10–0.13)	
	SS 3′	3.57 (2.98–3.78)	**<0.001**	100.00 (100.00–100.00)	0.317	0.11 (0.06–0.15)	0.668
	PI 3′	0.00 (0.00–0.00)		100.00 (100.00–100.00)		0.10 (0.05–0.27)	
	SS 5′	3.80 (3.38–4.04)	**<0.001**	100.00 (100.00–100.00)	0.509	0.10 (0.07–0.18)	0.087
	PI 5′	0.00 (0.00–0.00)		100.00 (100.00–100.00)		0.21 (0.07–0.68)	
**25–50 μm Polytxt®**	SS 1′	4.32 (4.16–5.04)	**<0.001**	100.00 (100.00–100.00)	**0.037**	0.03 (0.02–0.07)	**0.001**
	PI 1′	0.00 (0.00–0.00)		100.00 (98.08–100.00)		0.01 (0.00–0.03)	
	SS 3′	4.60 (4.25–5.22)	**<0.001**	100.00 (100.00–100.00)	0.903	0.06 (0.04–0.13)	**< 0.001**
	PI 3′	0.00 (0.00–0.00)		100.00 (100.00–100.00)		0.01 (0.01–0.02)	
	SS 5′	4.61 (4.51–5.20)	**<0.001**	100.00 (99.18–100.00)	0.146	0.04 (0.02–0.07)	**0.002**
	PI 5′	0.00 (0.00–0.00)		100.00 (90.83–100.00)		0.01 (0.01–0.02)	
**50–100 μm macrotextured**	SS 1′	3.84 (3.31–4.21)	**<0.001**	100.00 (100.00–100.00)	0.50	0.05 (0.03–0.07)	0.775
	PI 1′	0.00 (0.00–0.00)		100.00 (93.65–100.00)		0.05 (0.04–0.07)	
	SS 3′	3.82 (3.18–4.06)	**<0.001**	100.00 (99.38–100.00)	0.071	0.05 (0.03–0.10)	0.775
	PI 3′	0.00 (0.00–0.00)		99.45 (94.18–100.00)		0.05 (0.04–0.07)	
	SS 5′	3.70 (3.52–4.10)	**<0.001**	100.00 (95.78–100.00)	0.686	0.03 (0.02–0.08)	0.775
	PI 5′	0.00 (0.00–0.00)		100.00 (94.63–100.00)		0.05 (0.04–0.07)	

*SS, sterile saline; PI, povidone iodine; IQR, interquartile range; cfu, colony forming units; bacterial density, bacteria/μm^2^.*

*Values in bold represent statistical significance in favor of PI.

In general terms, the highest percentage reduction of living cells was only reached at PI5′ treatment in 25–50 μm Polytxt® implants (5.1%), which was not statistically nor clinically significant (Table 3, Figures 5A–C, and Supplementary Figures 11–13).

Regarding the reduction of bacterial density, we observed that, overall, 25–50 μm Polytxt® implants showed better average reduction rates at the three times of exposure with PI (PI1′: 73.3%, PI3′: 74.4%, and PI5′: 62.1%), reaching also statistical significance (0.001, < 0.001, 0.002). In addition, < 10 μm Polysmooth™ implants at PI1′ and PI3′ reached a reduction of 40.8% and 43.5%, respectively, but without showing statistical significance, except with PI1′ for *S. aureus* (*p* = 0.003) and with PI3′ for *S. epidermidis* (*p* = 0.009). Finally, 25 μm MESMO® implants showed both a clinical and statistically significant reduction of bacterial density in PI1′ treatment [median (IQR): PI1′ 0.11 (0.10–0.13) vs. SS 1′ 0.17 (0.14–0.19), *p* = 0.022, 29.4% reduction] (Table 3, Figures 5A–C, and Supplementary Figures 11–13).

#### Comparison

Data of the comparison between each type of Polytech® implants are detailed in Table 4. Generally, all type of implants, except the < 10 μm Polysmooth™, showed higher reduction of cfu counts after PI treatment.

**TABLE 4:** Comparative results for povidone iodine at three times of exposure according to Polytech® implants.

	Treatment	Median (IQR) log ufc/ml	*p* *	Median (IQR)% live cells	*p* *	Median (IQR)% bacterial density	*P* *
	**PI 1′ < 10 μm Polysmooth™**	0.00 (0.00–2.23)	**0.008**	100.00 (100.00–100.00)	0.749	0.12 (0.07–0.29)	**< 0.001**
	**PI 1′ 25–50 μm Polytxt®**	0.00 (0.00–0.00)		100.00 (98.08–100.00)		0.01 (0.00–0.03)	
**<10 μm Polysmooth™**	**PI 3′<10 μm Polysmooth™**	0.00 (0.00–1.72)	**0.008**	100.00 (100.00–100.00)	0.752	0.12 (0.04–0.20)	**<0.001**
**vs.**	**PI 3′ 25–50 μm Polytxt®**	0.00 (0.00–0.00)		100.00 (100.00–100.00)		0.01 (0.00–0.02)	
**25–50 μm Polytxt®**	**PI 5′ < 10 μm Polysmooth™**	0.00 (0.00–1.51)	**0.008**	100.00 (89.90–100.00)	0.868	0.19 (0.12–0.30)	**<0.001**
	**PI 5′ 25–50 μm Polytxt®**	0.00 (0.00–0.00)		100.00 (90.83–100.00)		0.01 (0.01–0.02)	
	**PI 1′ < 10 μm Polysmooth™**	0.00 (0.00–2.23)	**0.008**	100.00 (100.00–100.00)	0.447	0.12 (0.07–0.29)	**0.010**
	**PI 1′ 50–100 μm macrotextured**	0.00 (0.00–0.00)		100.00 (93.63–100.00)		0.05 (0.04–0.07)	
**<10 μm Polysmooth™**	**PI 3′<10 μm Polysmooth™**	0.00 (0.00–1.72)	**0.008**	100.00 (100.00–100.00)	**0.027**	0.12 (0.04–0.20)	0.153
**vs.**	**PI 3′ 50–100 μm macrotextured**	0.00 (0.00–0.00)		99.45 (94.18–100.00)		0.05 (0.04–0.07)	
**50–100 μm macrotextured**	**PI 5′ < 10 μm Polysmooth™**	0.00 (0.00–1.51)	**0.008**	100.00 (89.90–100.00)	0.883	0.19 (0.12–0.30)	**<0.001**
	**PI 5′ 50–100 μm macrotextured**	0.00 (0.00–0.00)		100.00 (94.63–100.00)		0.05 (0.04–0.07)	
	**PI 1′ < 10 μm Polysmooth™**	0.00 (0.00–2.23)	**0.008**	100.00 (100.00–100.00)	0.903	0.12 (0.07–0.29)	1
	**PI 1′ 25 μm MESMO®**	0.00 (0.00–0.00)		100.00 (100.00–100.00)		0.11 (0.10–0.13)	
**<10 μm Polysmooth™**	**PI 3′<10 μm Polysmooth™**	0.00 (0.00–1.72)	0.031	100.00 (100.00–100.00)	0.509	0.12 (0.04–0.20)	0.775
**vs.**	**PI 3′ 25 μm MESMO®**	0.00 (0.00–0.00)		100.00 (100.00–100.00)		0.10 (0.05–0.27)	
**25 μm MESMO®**	**PI 5′ < 10 μm Polysmooth™**	0.00 (0.00–1.51)	**0.008**	100.00 (89.90–100.00)	0.082	0.19 (0.12–0.30)	1
	**PI 5′ 25 μm MESMO®**	0.00 (0.00–0.00)		100.00 (100.00–100.00)		0.21 (0.12–0.30)	
	**PI 1′ 50–100 μm macrotextured**	0.00 (0.00–0.00)	1	100.00 (93.65–100.00)	0.677	0.05 (0.04–0.07)	**<0.001**
	**PI 1′ 25–50 μm Polytxt®**	0.00 (0.00–0.00)		100.00 (98.08–100.00)		0.01 (0.00–0.03)	
**50–100 μm macrotextured**	**PI 3′ 50–100 μm macrotextured**	0.00 (0.00–0.00)	1	99.45 (94.18–100.00)	0.048	0.05 (0.04–0.07)	**<0.001**
**vs.**	**PI 3′ 25–50 μm Polytxt®**	0.00 (0.00–0.00)		100.00 (100.00–100.00)		0.01 (0.00–0.02)	
**25–50 μm Polytxt®**	**PI 5′ 50–100 μm macrotextured**	0.00 (0.00–0.00)	1	100.00 (94.63–100.00)	0.679	0.05 (0.04–0.07)	**<0.001**
	**PI 5′ 25–50 μm Polytxt®**	0.00 (0.00–0.00)		100.00 (90.83–100.00)		0.01 (0.01–0.02)	
	**PI 1′ 25 μm MESMO®**	0.00 (0.00–0.00)	1	100.00 (100.00–100.00)	0.492	0.11 (0.10–0.13)	**<0.001**
	**PI 1′ 25–50 μm Polytxt®**	0.00 (0.00–0.00)		100.00 (98.08–100.00)		0.01 (0.00–0.03)	
**25 μm MESMO®**	**PI 3′ 25 μm MESMO®**	0.00 (0.00–0.00)	0.317	100.00 (100.00–100.00)	0.258	0.10 (0.05–0.27)	**<0.001**
**vs.**	**PI 3′ 25–50 μm Polytxt®**	0.00 (0.00–0.00)		100.00 (100.00–100.00)		0.01 (0.01–0.02)	
**25–50 μm Polytxt®**	**PI 5′ 25 μm MESMO®**	0.00 (0.00–0.00)	1	100.00 (100.00–100.00)	0.046	0.21 (0.07–0.68)	**<0.001**
	**PI 5′25–50 μm Polytxt®**	0.00 (0.00–0.00)		100.00 (90.83–100.00)		0.01 (0.01–0.02)	
	**PI 1′ 50–100 μm macrotextured**	0.00 (0.00–0.00)	1	100.00 (93.65–100.00)	0.268	0.05 (0.04–0.07)	**<0.001**
	**PI 1′ 25 μm MESMO®**	0.00 (0.00–0.00)		100.00 (100.00–100.00)		0.11 (0.10–0.13)	
**50–100 μm macrotextured**	**PI 3′ 50–100 μm macrotextured**	0.00 (0.00–0.00)	0.317	99.45 (94.18–100.00)	**0.002**	0.05 (0.04–0.07)	**0.063**
**vs.**	**PI 3′ 25 μm MESMO®**	0.00 (0.00–0.00)		100.00 (100.00–100.00)		0.10 (0.05–0.27)	
**25 μm MESMO®**	**PI 5′ 50–100 μm macrotextured**	0.00 (0.00–0.00)	1	100.00 (94.63–100.00)	0.058	0.05 (0.04–0.07)	**0.001**
	**PI 5′ 25 μm MESMO®**	0.00 (0.00–0.00)		100.00 (100.00–100.00)		0.21 (0.07–0.68)	

*PI, povidone iodine; IQR, interquartile range; cfu, colony forming units; bacterial density, bacteria/μm^2^.*

*Values in bold represent statistical significance.

A reduction in the median of living cells was imperceptible between the four types of implants, with statistical significance found occasionally.

Unlike what was observed in Mentor^®^, < 10 μm Polysmooth™ implants showed worse median reduction of bacterial density in the biofilm treated with PI at any time of exposure compared to 25 μm MESMO^®^, 25–50 μm Polytxt® and 50–100 μm macrotextured (Supplementary Figures 4–7).

As well as in Mentor, the degree of PI absorption was different depending on the type of Polytech® implants, being 25–50 μm Polytxt® implants the one with the highest degree of absorption (Figures 4D–G).

## Discussion

Given the fact that hitherto the exact cause for capsular contraction has not yet been determined sufficiently, the biofilm hypothesis is one of the major corner stones to explain fibroblast proliferation. Chronic inflammation is somehow considered to play a role in this entity and hence numerous authors have tried to circumvent biofilm occurrence utilizing various disinfectants or antibiotic solutions, applied directly to implants, and into the implant pockets (Dang et al., 2020; Jewell et al., 2021; Nai et al., 2021).

In the spring of 2000, the US Food and Drug Administration (FDA) issued a ban on the use of Betadine (Purdue Frederick, Stamford, Connecticut) with BI due to concerns about an adverse effect on shell integrity that could lead to implant deflation or rupture (Wiener, 2013). In 2017 the United States FDA reviewed and approved a request by a BI manufacturer for a change in directions for use that removed warnings regarding the use of Betadine (PI) 10% solution. It has been proposed that Betadine would not have a significantly negative impact on shell integrity but might help influence to reduce capsular contraction (Wiener, 2007).

Nevertheless, meanwhile it has been shown in an *in vitro* study that in principle Betadine is not harmful to silicone implant surfaces when administered in a clinically usual time frame (Schmitz et al., 2019).

Despite several preventive measures exist to reduce BI infection, being the irrigation of the implant with local antiseptics one of the most widely used (Chopra et al., 2017; Drinane et al., 2017; Banerjee and Featherstone, 2019; Carvajal et al., 2019), there is no consensus on which antiseptic, at what dose, and for how long it should be used to obtain optimal prevention. In a recent study by Ngaage et al. (2020) it was shown that 10% PI had the best results compared to other antiseptics when applied for 30 min on smooth implants colonized by *S. aureus* and *S. epidermidis*. However, since that time of exposure was too long to be applied in the real clinical practice and since implants with different degrees of texture are used in Spain, we proceeded to optimize the efficacy of PI at 3 shorter exposure times, as well as studying different textures using both conventional culture and CLSM.

In general, we observed that PI at the 3 exposure times was able to significantly reduce (mostly inhibit) cfu counts after culture of the sonicate from disks implants, regardless of the microorganism tested, compared to the control group treated with SS. All textures, except for smooth implants had sterile cultures (100% reduction) from the disks sonicate (mean percentage reduction of smooth implants ranged between 51.9 and 82.9%). However, these differences could not be demonstrated when analyzing the percentage of living cells. Although mean reduction ranged from 0.1 to 14.4%, it was not enough to reach clinical impact (>25%).

Regarding bacterial density, Mentor® implants treated with PI showed an augmentation in number of bacteria/μm^2^ (except for 10 μm—smooth). However, Polytech® implants (except for macrotextured 50–100 μm) showed a significant mean reduction of bacterial density, with 25–50 μm Polytxt® the one that showed better reduction rates at each PI exposure time (PI1′: 73.3%, PI3′: 74.4%, and PI5′: 62.1%).

A possible explanation for this lack of correlation between reduction of cfu counts and cell viability/bacterial density could be that the cells lost their ability to grow in microbial culture and, therefore were not recoverable, the so-called “viable but not culturable cells.” The impact of this cell state is still under debate, although several authors favor the theory that these cells are in the pre-death phase, so they are still viable but not recoverable. Despite having all the nutrients necessary for their growth, they are not able to grow and sooner or later they will die (Li et al., 2014; Zhao et al., 2017).

Regarding the optimal exposure time of PI to reduce bacterial biofilm, we found no differences between the 3 times tested. Although the recent study by Ngaage et al. (2020) showed that 10% PI was effective when breast implants disks were left immersed for 30 min, this time is neither viable nor feasible to apply in clinical practice, since it is not possible to keep the patient in the operating room waiting 30 min for the solution to act. Therefore, according to our data, given that the variable that showed the greatest reduction rates regardless of time exposure tested was cfu count, we consider that there is no relationship between PI exposure time and its effect, which allows us to use it in clinical practice for < 30 min of exposure. In our opinion, optimization of the efficacy of PI is not so much due to the exposure time, but rather to its penetration capacity on the silicone material of the implants. In the recent study by Barnea et al. (2018), given that the surface of BI is hydrophobic, they pre-treat the implants with plasma to reverse their hydrophobic to hydrophilic condition, and their results are spectacular after subsequent immersion of the implants in the PI solution for only 5 s. Despite they demonstrated that treating BI with plasma to improve implant permeability to PI, our study is the first to assess that PI efficacy against Staphylococcal biofilms is not dependent of the time of exposure but of the implant surface. Therefore, this would support our hypothesis that the effectiveness does not depend so much on the exposure time but on the penetration of the solution, as it can be observed in Figure 4, where depending on the implants’ texture, the penetration ability of PI is different. We suggest that what Barnea et al. (2018) demonstrated is a promising sept for Plastic Surgery that needs to be further assess in clinical studies.

This is also related to the possible differences in the effectiveness of PI according to the texture of the implants. Previous studies have shown that colonization occur more frequently in smooth implants in comparison with macrotextured implants (Ersek, 1991; Wong et al., 2006). We have been able to confirm these results, as the implants with the least reduction of colonization degree were the smooth ones. However, contrary to what might be expected, the density of the biofilm in the Mentor® smooth texture (10 μm—smooth) was significantly lower than in the microtextured (28 μm Siltex™ and 36 μm CPG™ implants). Conversely, in the latter, although the reduction in biofilm density was lower, the reduction in cfu count per culture was greater. This could be explained by the fact that cells adhere more to the surface of smooth implants, which have less biofilm. With this adherence, cells are harder to detach, and treatment with PI would not optimally access and eliminate those cells. The opposite would occur in the microtextured ones, where the biofilm is thicker and the cells are less adhered and detach more easily, and PI can act more effectively (Li et al., 2014; Barnea et al., 2018; Ngaage et al., 2020). This was not observed in Polytech® implants.

Despite demonstrating good results with the application of PI during 1′ at BI, future studies are needed to validate the recent application of plasma to better penetrate silicone, as well as to test PI efficacy in other microorganisms, such as *Cutibacterium. acnes*, and with other types of implants. Moreover, since there is a complex bacterial ecosystem surrounding BI, further research is needed regarding the role of microbioma of the biofilms in the pathogenesis of BI infection and capsular contracture (Cook et al., 2021; Crowe and Simister, 2021).

One the main limitations of the study is that, in order to mimic the clinical scenario, we did not rinse silicone disks after the adsorption step and were immediately transferred to the bacterial solution, which may have influenced PI amount on surfaces. Moreover, regarding some concerns for tissue toxicity and cellular damage with PI, we used betadine solution (not soap) and the relative cytotoxicity of betadine is considered a beneficial effect around a breast implant, given that it would decrease the proliferation of fibroblasts (related to the periprosthetic capsule), which would imply a lower incidence of capsular contracture. This is one of the beneficial effects of betadine that is intended to be demonstrated in a further subsequent *in vivo* study in which we will assess not only the colonization of the implants upon explanting, but also the infection rates and the state of the resulting periprosthetic capsule after placing miniature implants in rodents after irrigation with betadine.

Our data need to be validated by investigating different microorganisms and with various other types of implants. It remains also true that the complex bacterial system of biofilms (quorum sensing) is by far not sufficiently understood and needs to be further investigated to better specify the optimal attack point against biofilms and capsular contraction.

## Conclusion

We demonstrated that PI was able to inhibit bacterial growth applied on the surface of breast implants regardless of the exposure time. However, no significant reduction on living cells or bacterial density was observed. This lack of correlation may be caused by differences in texture that directly affect PI absorption.

## Data Availability Statement

The original contributions presented in the study are included in the article/Supplementary Material, further inquiries can be directed to the corresponding author/s.

## Author Contributions

MG and ÁG-R were responsible for the organization and coordination of the trial. RP-C, JL, PM, and MG were the chief investigator and responsible for the data analysis. BF-I, MD-N, GI, AR, RH, RC, RP-C, and ÃI developed the trial design and data collection. All authors contributed to the writing of the final manuscript.

## Conflict of Interest

The authors declare that the research was conducted in the absence of any commercial or financial relationships that could be construed as a potential conflict of interest.

## Publisher’s Note

All claims expressed in this article are solely those of the authors and do not necessarily represent those of their affiliated organizations, or those of the publisher, the editors and the reviewers. Any product that may be evaluated in this article, or claim that may be made by its manufacturer, is not guaranteed or endorsed by the publisher.
